# A self-updating road map of The Cancer Genome Atlas

**DOI:** 10.1093/bioinformatics/btt141

**Published:** 2013-04-17

**Authors:** David E. Robbins, Alexander Grüneberg, Helena F. Deus, Murat M. Tanik, Jonas S. Almeida

**Affiliations:** ^1^Division of Informatics, Department of Pathology, University of Alabama at Birmingham, Birmingham, AL 35233-7331, USA, ^2^Department of Electrical and Computer Engineering, University of Alabama at Birmingham, Birmingham, AL 35249, USA and ^3^Digital Enterprise Research Institute (DERI), National University of Ireland, Galway, Ireland

## Abstract

**Motivation:** Since 2011, The Cancer Genome Atlas’ (TCGA) files have been accessible through HTTP from a public site, creating entirely new possibilities for cancer informatics by enhancing data discovery and retrieval. Significantly, these enhancements enable the reporting of analysis results that can be fully traced to and reproduced using their source data. However, to realize this possibility, a continually updated road map of files in the TCGA is required. Creation of such a road map represents a significant data modeling challenge, due to the size and fluidity of this resource: each of the 33 cancer types is instantiated in only partially overlapping sets of analytical platforms, while the number of data files available doubles approximately every 7 months.

**Results:** We developed an engine to index and annotate the TCGA files, relying exclusively on third-generation web technologies (Web 3.0). Specifically, this engine uses JavaScript in conjunction with the World Wide Web Consortium’s (W3C) Resource Description Framework (RDF), and SPARQL, the query language for RDF, to capture metadata of files in the TCGA open-access HTTP directory. The resulting index may be queried using SPARQL, and enables file-level provenance annotations as well as discovery of arbitrary subsets of files, based on their metadata, using web standard languages. In turn, these abilities enhance the reproducibility and distribution of novel results delivered as elements of a web-based computational ecosystem. The development of the TCGA Roadmap engine was found to provide specific clues about how biomedical big data initiatives should be exposed as public resources for exploratory analysis, data mining and reproducible research. These specific design elements align with the concept of knowledge reengineering and represent a sharp departure from top-down approaches in grid initiatives such as CaBIG. They also present a much more interoperable and reproducible alternative to the still pervasive use of data portals.

**Availability:** A prepared dashboard, including links to source code and a SPARQL endpoint, is available at http://bit.ly/TCGARoadmap. A video tutorial is available at http://bit.ly/TCGARoadmapTutorial.

**Contact:** robbinsd@uab.edu

## 1 INTRODUCTION

The Cancer Genome Atlas (TCGA) is a joint project of the National Cancer Institute (NCI) and the National Human Genome Research Institute (NHGRI) to comprehensively apply genome analysis technology to the study of the biomolecular basis of cancer (NCI Wiki, 2011). Concretely, this project has analyzed tumor and normal samples from over 6000 patients, which resulted in the collection and public availability of 37 types of genomic and clinical data for 33 cancers. These data types include gene expression, single-nucleotide polymorphism, miRNA, copy number, DNA methylation and somatic mutations, along with tissue slide images and clinical outcomes. Since 2011, this data has been available via an HTTP repository (http://1.usa.gov/OTmJac). This report details the creation and study of a road map of the TCGA’s HTTP repository, created to enable the use of this unprecedented biomolecular data resource in the creation of Web 3.0 applications, and enhance the reproducibility of biomolecular research delivered as elements of a computational ecosystem.

In a previous report (Deus *et al.*, 2010), the authors have identified a Resource Description Framework (RDF) data model describing the contents of TCGA file repository. The resulting RDF map of the TCGA contents is available (http://rdf.s3db.googlecode.com/hg/TCGA.rdf), and can be efficiently traversed by a SPARQL engine to quickly discover which files document results that satisfy any number of the constraints recognized by the model. For example, as illustrated in a webcast accompanying that manuscript (http://youtu.be/BI5bf-taGU4), one could identify which files describe patients from a specific cancer center that provided samples that were profiled for DNA copy number variation.

Since then, the TCGA initiative has greatly expanded the number of patients, the volume of data and the diversity of analytical platforms used. The complexity of the data available has also increased: each cancer type has a unique, only partially overlapping, subset of available data types. More importantly, in 2011, there was a momentous change in the level of data interoperability of the TCGA data repository: data files are now available directly through HTTP calls to a central directory, located at http://1.usa.gov/OTmJac. This opens entirely new opportunities for interactive reproducible data analysis and visualization.

Indeed, as the TCGA and other collaborative initiatives of this scope evolve and expand, it is not reasonable to expect that they will conform to a narrowly defined format or structure for the duration of the initiative. As a consequence, an attempt to use the 2010 RDF road map linked above to traverse the current contents of the TCGA initiative is likely to produce a significant number of unresolvable links to data files. Therefore, achieving persistent interoperability of the TCGA initiative requires a different data modeling approach, one that relies on a data model with a versioned data file road map engine. In the past 2 years, the third generation of web technologies (Hendler, 2009) matured to the extent that they now provide the foundation for multiple big data resources, such as those integrated by Data.gov (Hendler *et al.*, 2012). As a consequence, the opportunity to develop versioned road maps, programmatically interoperable via SPARQL is now at hand.

Simultaneous to the expansion of the TCGA, the tooling required for enabling computational ecosystems for data-driven medical genomics (Almeida, 2010) is maturing rapidly, to the point that tools operating within and providing such ecosystems are beginning to appear (Almeida *et al.*, 2012b). The concern that the web browser is computationally inefficient for advanced numerical procedures has also been amply overcome, as we found when identifying sequence analysis procedures making use of the MapReduce (Dean and Ghemawat, 2008) distributed computing template (Almeida *et al.*, 2012a; Vinga *et al.*, 2012).

A core requirement of applications operating within such a computational ecosystem is the ability to discover, access and analyze subsets of large data services, as underscored by the recent doubling of the number of recognized breast cancer subtypes (Curtis *et al.*, 2012). Although studies as those reported in (Curtis *et al.*, 2012), and other large-scale integrative analyses using the TCGA (Cooper *et al.*, 2012; Setty *et al.*, 2012; TCGA Research Network, 2008; TCGA Research Network, 2011; Zeeberg *et al.*, 2012), themselves make use of broad datasets, their results are often the starting point for further study of the numerous biomolecular bases for tumorigenesis. Navigating this entangled web of linked data becomes even more challenging in the context of personalizing care, which requires (i) the identification of the reference sources most relevant to a given patient and (ii) delivery of computational tools for personalized analysis of a subset of the data within the same computational environment used for data discovery. A recent use of data resources in the TCGA for morphological analysis (Cooper *et al.*, 2012) underscores the increasing use of the TCGA as a universal cancer reference, not just for genomics information, but for full patient profiles.

The need to navigate through data portals, such as reported in (Zhang *et al*., 2011a) or download bulk data prevents programmatic exploration of the contents of the TCGA, hampering the leveraging of this wealth of data in point-of-care scenarios. Conversely, the ability to programmatically identify which of the ½ million data files in the TCGA are relevant to a particular problem would enable not only large-scale comprehensive study of cancer genomes, but also the creation of tools capable of real time, on the fly analysis and presentation (for an example, see http://bit.ly/TCGA-RPPA).

Recent calls for reproducible research (Baggerly and Berry, 2011) underscore the need for novel algorithms and analyses delivered in the context of such computational ecosystems to provide provenance information on the data used. Although providing links to bulk data, as in (Zeeberg *et al.*, 2012), or referencing all data in a data portal (Setty *et al.*, 2012), fulfills this requirement for traditionally reported research, the ability to annotate particular results with the precise data file, from the original source, that led to the result, would be a better solution. Such a solution would deliver immediately reproducible results as elements of a computational ecosystem. Detailed provenance information and full traceability of all data and analytical components becomes even more critical in an emerging big-data-driven knowledge reengineering scenario (Hoekstra, 2011; Robbins and Chiesa, 2011).

The need for fully reproducible analytical results from patient data, which in turn requires the ability to traverse the original data repository efficiently, has begun to receive attention. For example, the reference ClinicalTrials.gov (http://clinicaltrials.gov/) initiative has recently overlaid its relational databases with a SPARQL engine (D2RQ, http://d2rq.org/), which can be accessed at http://static.linkedct.org/snorql/. This approach, however, will not necessarily work for the TCGA because of its ontological fluidity, which invites a knowledge reengineering approach. The TCGA Roadmap described in this report leverages this approach to deliver a framework for the efficient traversal of the TCGA’s open-access data.

## 2 METHODS

The road map engine software developed to represent resources in the TCGA as RDF triples was written using JavaScript (ECMAScript 5th ed.), and run using NodeJS (http://nodejs.org/). All source code was kept under revision control, and is available at https://github.com/tcga/Roadmap/.

RDF triples were stored in an AllegroGraph 4.9 database (by Franz Inc.), which was interacted with using SPARQL 1.1 over the HTTP interface provided. This database is publicly available for read access and SPARQL queries at http://bit.ly/TCGARoadmap.

Only data available in the TCGA’s open-access HTTP repository (http://1.usa.gov/OTmJac) was included in this road map. This decision was made to enable the widest possible use of the tool.

Reported figures describing the data in the road map were generated using JavaScript, Google Chart Tools (https://developers.google.com/chart/) and Data-Driven Documents (D3) (Bostock *et al.*, 2011). Interactive automatically updating versions of these figures, including the source code, may be accessed at http://bit.ly/TCGARoadmap.

## 3 RESULTS

In large data-gathering exercises, such as the TCGA, the degree of conservation in the structure of the data decreases with distance to the biological signal. In the case of the TCGA, this effect is manifest in the increased fluidity of metadata about files (e.g. their location, the protocols by which they are accessed) relative to the contents of those files, which remains static. To capture this fluidity in a queryable fashion, the TCGA’s open-access HTTP site is scraped into a collection of RDF triples describing the locations of, and relationships between, individual files.

### 3.1 Schema

The TCGA’s open-access HTTP site represents its data in a directory tree structure, with each level corresponding to a different element of the metadata about the files contained at the extremities of the tree (NCI Wiki, 2011). Using a simple schema (Table 1), metadata for each resource accessible in this tree structure is recorded in RDF.

**Table 1.** Simple RDF schema for describing TCGA resources

Property	Range	Description
tcga:url	HTTP URL literals	The URL of the resource in the TCGA
rdfs:label	String literals	Name of the resource in the TCGA
rdf:type	tcga:DiseaseStudy	Type of the resource, determined by its depth in the directory tree structure
	tcga:CenterType	
	tcga:CenterDomain	
	tcga:Platform	
	tcga:DataType	
	tcga:Archive	
	tcga:File	
tcga:firstSeen	Date literal	First date a resource was scraped
tcga:lastSeen	Date literal	Date of the most recent scrape that included the resource
tcga:lastModified	Date literal	Unix modified date of the resource, as given by the TCGA

*Note*: All properties have the domain of TCGA entities. TCGA entities are identified by UUID, and have the form tcga:<uuid> (e.g. tcga:8a4e160d-92f3-448d-aa3d-ffe24c34b342). For files, the modified date represents the last time the file was ‘touched’, or its contents changed. For folders (e.g. a folder representing a disease study), the modified date represents the last time the set of immediate children in the directory tree changed (e.g. a file was added or removed).

**Fig. 1.**
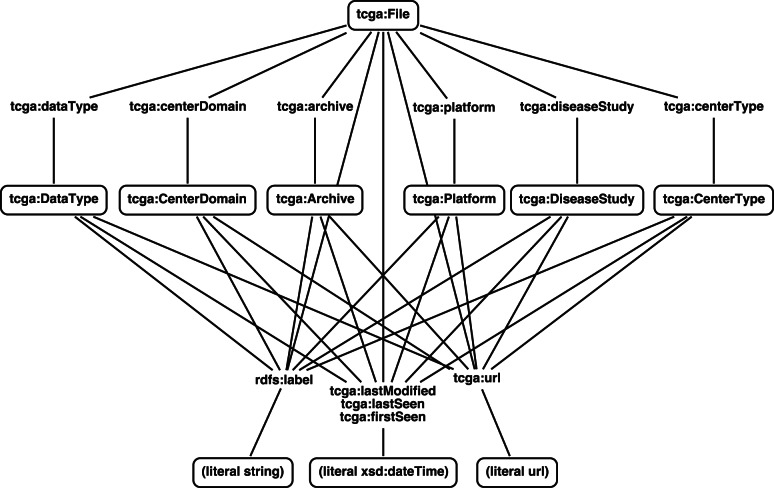
Class diagram of the schema used to represent TCGA files in RDF. Each class may have the *tcga:lastSeen*, *tcga:lastModified*, *tcga:firstSeen*, *tcga:url* and *rdfs:label* properties. In addition to these, *tcga:File* resources have properties indicating the particular instances of the other resource types they belong to, such as the *tcga:Archive* resource they are contained in. The properties for linking *tcga:Files* to other classes are generated by using a lower case version of the class name (e.g. *tcga:archive* links a *tcga:File* and a *tcga:Archive*)

Figure 1 provides a class diagram representation of this schema. Each resource is assigned a universally unique identifier (UUID) for use in the RDF store. Each type of resource in the TCGA has a human readable name (label), a reference to its origin (URL) and a last-modified date captured, along with the first and last dates it was seen by the road map engine. In addition, data files in the TCGA also have links to their respective archives, data types, platforms, centers, center types and disease studies.

**Fig. 2.**
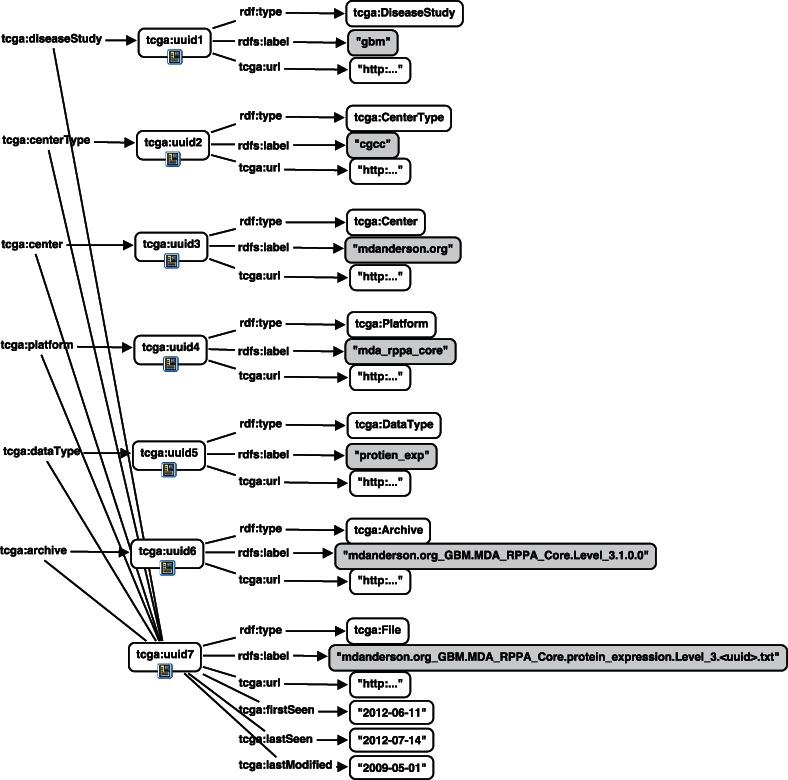
Example representation of metadata about a file in the TCGA using our schema. This portion of the RDF graph shows a file for the platform *mda_rppa_core*, from M.D. Anderson (mdanderson.org), which is a cancer genome characterization center (cgcc) in the glioblastoma disease study (*gbm*)

An example of a file instance in this schema is given in Figure 2, representing a file from the *mda_rppa_core* platform from mdanderson.org, a cancer genome characterization center (*cgcc*) studying glioblastoma (*gbm*). Note that to conserve space, the *tcga:lastSeen*, *tcga:lastModified* and *tcga:firstSeen* properties are only shown for the *tcga:File* resource, although they exist for all types.

### 3.2 Scraping and storage

**Fig. 3.**
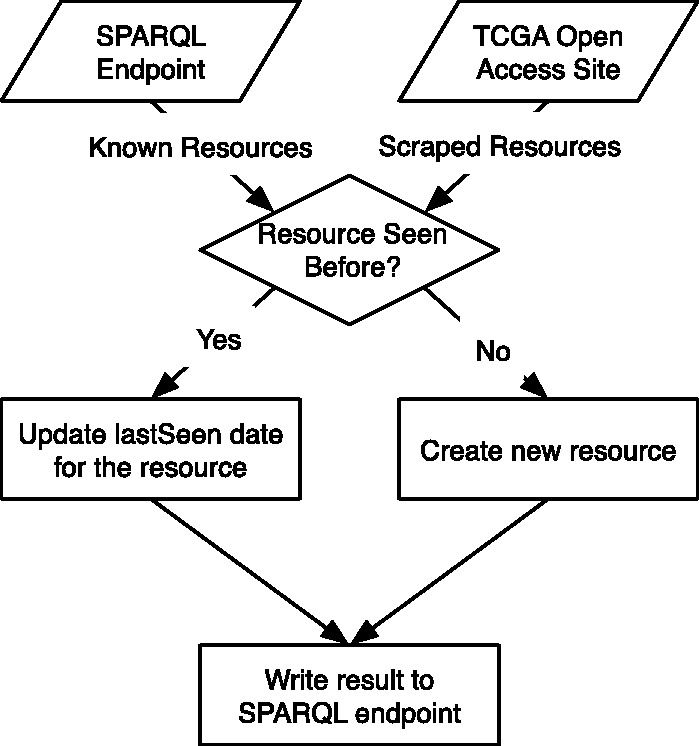
Flowchart representation of the algorithm used to scrape and update the TCGA open-access HTTP site into an RDF road map via a SPARQL endpoint

Figure 3 provides a flowchart representation of the algorithm used to scrape and update the RDF representation of the TCGA files. After retrieving a list of known resources from the SPARQL endpoint, the road map engine begins retrieving new resources from the TCGA’s open-access HTTP site. If a retrieved resource exists in the list of known resources, its *tcga:lastSeen* date is updated. If the retrieved resource does not exist in the list of known resources, a new UUID and triples representing the metadata for the resource are generated. In either case, the results are written to the SPARQL endpoint. If a resource is any type other than *tcga:File*, each of its child entities are then also added, recursively, via depth-first traversal of the tree structure.

The road map is kept up to date by periodically rerunning the road map engine again, which performs the same depth-first traversal. This process is currently triggered once every 24 h by an automated script, but may be triggered manually.

### 3.3 TCGA Roadmap dashboard

It is noteworthy that all figures in this portion of the report may be regenerated at http://bit.ly/TCGARoadmap, providing instant, interactive reproducibility of these results. As a result of the ability to efficiently traverse the metadata on the entire open-access TCGA repository, an online dashboard was deployed to illustrate the advantages of having a completely decoupled presentation layer. As discussed later, this is in contrast to the data portal approach that still dominates the delivery of patient-derived biomolecular data.

**Fig. 4.**
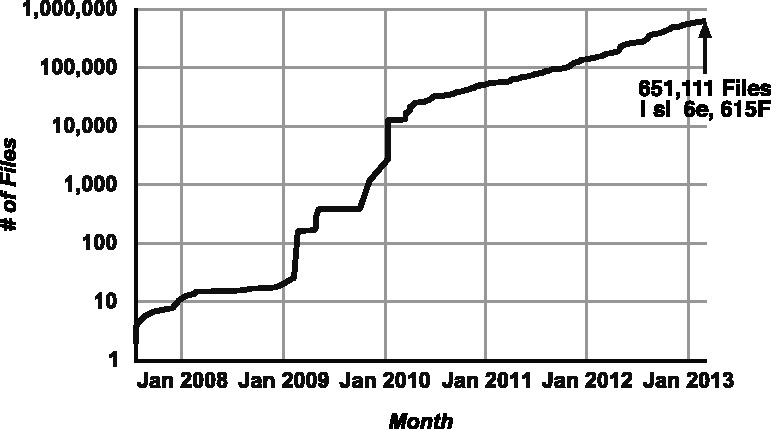
Snapshot of a dashboard element showing the logarithmic progression of the number of files available to the public in the TCGA, counted using the SPARQL endpoint here reported, showing a sustained doubling every 7 months since March 2010, and now over ½ million individual files in the public domain. The query used to generate the results may be accessed at http://bit.ly/FilesByDate, and a live automatically updating version of this figure is available at http://bit.ly/TCGARoadmap

Since our initial report in September 2010, the TCGA has sustained a doubling in size every 7 months, as measured by the number of files publically available (Figure 4). As a result of this growth, over ½ million files are now available in the public domain.

**Fig. 5.**
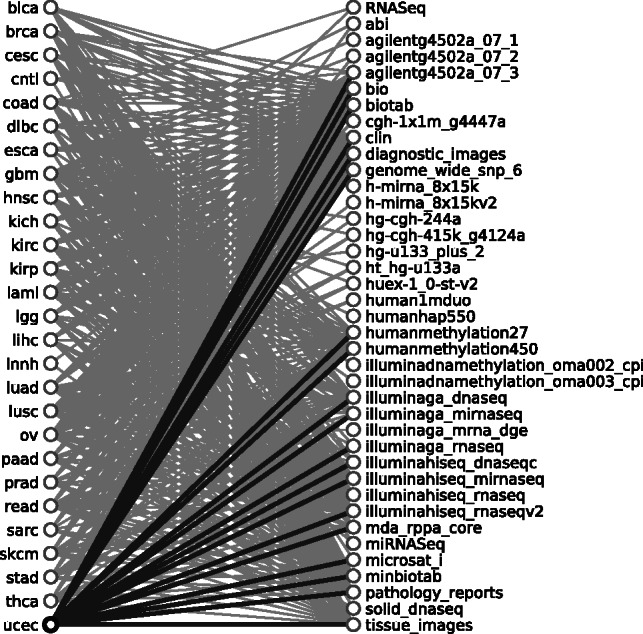
Snapshot of a dashboard element showing the results of querying the TCGA contents for relationships between platforms and disease studies, shown here as a bipartite graph. In this figure, lines between disease studies (on the left) and data analysis platforms (on the right) indicate that the disease study contains files generated by the linked platform. Uterine corpus endometrioid carcinoma (coded ucec in TCGA) is highlighted, as well as the lines for the 17 platforms used to process samples within that study. The query used to retrieve this data is available at http://bit.ly/PlatformsByDisease, with an interactive version of the visualization of the data available at http://bit.ly/TCGARoadmap

Simultaneous to growth in size, the publicly available data in the TCGA has grown in complexity. Samples within each disease study, representing the study of a particular tumor type, are analyzed by a variety of platforms: as many as 23 for ovarian serous cystadenocarcinoma (coded *ov* in TCGA), or as few as four for kidney chromophobe (coded *kich*). Visualizing the connections between disease studies and platforms as a bipartite graph (Figure 5) reveals the overlapping sets of platforms used within each disease study.

## 4 DISCUSSION

By capturing the date first seen, the date last seen and the date last modified, reproducible resolution of sets of files in the TCGA is enabled. Querying for all files first seen before a given date allows reproduction of an analysis done in the past, assuming none of the files have been removed. Similarly, reproducing analyses performed before the initial scrape of the TCGA can be accomplished by querying by last-modified date rather than date first seen.

Files that have been removed can be detected by looking for those with a last-seen date earlier than the latest last-seen date in the repository. Their existence in the repository indicates they at one time existed in the TCGA, while their out-of-date last-seen date indicates they were not present during a subsequent scrape.

### 4.1 Advances beyond our 2010 model

In (Deus *et al.*, 2010), we envisioned a hosted service depicting complex relationships between samples, patients and other higher order entities in the TCGA. This model was devised in the early stages of TCGA, before the organizational HTTP schema hosting it was stabilized. Furthermore, the relationships between the elements were represented using the S3DB Model (Almeida *et al.*, 2010) to enable the mix of public ‘omic’ results and private clinical parameters being generated at the M.D. Anderson Cancer Center. The ensuing public availability of clinical parameters by the TCGA Consortium made the usage of the S3DB model less relevant for these datasets. Furthermore, the older model did not anticipate the subsequent fluidity in the location metadata about the files with data expressing those complex relationships. This shortcoming made the model brittle, and subsequently unresolvable when the fluid metadata inevitably changed.

To advance past these shortcomings, our current model emphasizes the capture and representation of metadata about files, while leaving analysis of the contents of those files for other tools. As such, individual samples and patients are not modeled. Table 2 summarizes the key advances between this work and our 2010 efforts, reported in (Deus *et al.*, 2010).

**Table 2.** Simple RDF schema for describing TCGA resources

Property	2012	2010
Model	Simple	Complex^a^
Resolution	File/directory	Sample/patient
Environment	Web service	Web service
Size of TCGA	1/2 million files	50 000 files

^a^See Figure 3 of (Deus *et al.*, 2010).

### 4.2 Provenance tracking

One significant addition to the 2012 model for the TCGA datasets is the ability to enable provenance tracking for each of the data files, in addition to a data exploration tool, which was missing from the 2010 resource.

As an example, the 2010 model was centered on the sample—its main design focus was on capturing raw data about patients. As such, it was originally assumed that each patient had one raw data file for each type of data captured. In the 2010 model, each raw data file was captured using a dynamic link that returned the correct data file given a patient identifier and the data type, effectively making this the file’s Universal Resource Identifier (URI). In the new model, each raw data file is assigned a stable semantically neutral URI. In other words, the new URIs do not attempt to capture labels or qualifiers, but are instead generated as UUIDs. This shifts the provenance annotations from the URI syntax, where they cannot be used by semantic engines, to a properly RDF formalized set of provenance statements. In this new version, each file is extensively annotated with provenance information.

Provenance annotation is particularly relevant in biomedical datasets such as the TCGA: it is not uncommon for a new version of a patient raw data file to be submitted because the first version had bad quality or was not captured according to the predefined experimental protocols. As such, semantically neutral URI are the right option for the TCGA 2012 model as they ensure that our URI are ‘cool URI’ (Sauermann *et al.*, 2008): URI that do not change.

With this new methodology for URI naming in the TCGA Roadmap, we lay the groundwork for future efforts in which the data extracted from these files can be programmatically linked back to the files (or to a specific version of the files) via their URI. For example, a set or ‘batch’ of these files is typically included in a microarray analysis for the identification of disease biomarkers. These results can then be represented as RDF assertions, such as _*:geneX* _*:isBioMarkerFor* _*:LiverCancer*. The ability to report these resulting biomarkers with links to their source files using, as per W3C’s provenance notation working draft (Moreau *et al.*, 2012), the *prov:wasDerivedFrom* property, enables both the complete replication of the analysis results and provides a strong supporting argument for the RDF assertions generated by the data-processing step. This effectively enables the much needed back-tracking of data transformation and processing operations to the original raw data files (Baggerly and Berry, 2011; Deus *et al.*, 2012).

### 4.3 Sustainability and extensibility

Our road map represents a sustainable resource in that its generating engine is delivered as an open source tool, built on web standards (JavaScript, HTTP, RDF and SPARQL). The open source tools provided, in conjunction with a SPARQL endpoint, could be readily used to regenerate the road map reported here. Although the authors are committed to maintain a publicly available updated road map, these tools could be readily used to replace it should it cease to be maintained, or should a given user community wish to create their own independent road map.

The use of web standards and provision of a SPARQL endpoint also provide the basis for extensibility: RDF annotations allow the linking resources in the TCGA to related resources in other data stores, while SPARQL 1.1’s SERVICE queries allow federation of multiple data sources. For example, a given disease study in the TCGA may be annotated with links to resources corresponding to the disease under study in TCGA with descriptions of the disease in Diseaseome (http://thedatahub.org/dataset/fu-berlin-diseasome), or to clinical trials related to the disease in LinkedCT (http://linkedct.org/). Such annotations form the core of Linked Data approach (Berners-Lee, 2007), and add significantly to the usefulness of a big data resource.

### 4.4 Exposure requirements for public big data resources

As many others, we expect most public big data resources will eventually be provided as 5-star linked data (Berners-Lee, 2007). Data provided in this format does not require additional parsing before integration and knowledge reengineering (Hoekstra, 2011) in a Web 3.0 computational ecosystem, ensuring maximum return on public investment by enabling the widest possible use of a public big data resource. As discussed in the section on sustainability and extensibility, annotating a resource as linked data enables the extension of a given dataset through deep links into another dataset, defining a distributed but coherent global data space (Heath and Bizer, 2011).

The road map engine described in this report demonstrates the sufficiency of the first 3-stars in (Berners-Lee, 2007) (availability on the web under an open license, machine readability and non-proprietary format) as a minimal foundation, on which the community of users of the data may freely build the remaining elements (RDF representations, and linking annotations of that RDF). Thus, availability on the web under an open license, machine readability and use of non-proprietary formats form the core exposure requirements for public big data resources. Even within these three minimal requirements, degrees of efficiency in exposure and access exist. The necessity, for instance, of continually re-scraping the open-access HTTP directory provided by the TCGA to ensure the road map remains up to date is precipitated by the lack of a readily machine-digestible index. This necessity, in turn, could place unnecessary burdens, in the form of excessive HTTP requests per second, on the NIH servers hosting the TCGA data, especially if the community begins to create multiple duplicate road maps of the data.

Really Simple Syndication (RSS) feeds provided by the NIH (https://list.nih.gov/cgi-bin/wa.exe?A0=tcga-data-l) represent a possible solution based on web standards. However, at the time of this reporting, they syndicate only additions and maintenance announcements; deletions would still need to be scraped from the public HTTP directory. A second solution could take the form of providing a simple index in the form of a file list, such as generated by the Unix command *ls -Rl>index.txt* (which directs the creation of a list of all files and directories, recursively, and stores that list in a file named index.txt) at the root level of the directory. Providing such a list, containing files, their respective directories and metadata such as last-modified date, would also allow the maintenance of a road map based on a single HTTP request, rather than the currently required tens-of-thousands.

### 4.5 Summary of technical recommendations for biomedical big data hosting

The development and study of the self-updated SPARQL endpoint to support TCGA files discovery described here suggests that in addition to Linked Data standard recommendations (Berners-Lee, 2007), three additional features should be considered. These three features are specifically driven by concerns with interoperability and reproducibility:

1. URLs should be UUIDs, such that content is identified accurately. Editing the contents of a resource should correspond to the creation of a new resource, with a new URL, with the updated content.
2. Cross-Origin Resource Sharing (CORS) should be enabled, allowing access to data resources within the browser. This would be preferred to other approaches, such as the use of JSONP, because it allows for the use of pointers to retrieve individual lines of text within the file, further driving flexibility in data access.
3. A machine accessible log or index should be provided. As detailed in the previous section, scraping file lists as we did will eventually become unsustainable. A better option would be to include an index or change log of the resources made available. Ideally, this log will be accessible in the same place as the data; making consumers of the data search for separate mailing lists or syndication feeds represents a suboptimal solution.

### 4.6 Relationship to TCGA data portals

The indexing and road-mapping approach described in this report advances beyond the use of data portals in its adherence to Web 3.0 standards, rather than the *ad hoc* creation of query Domain-Specific Languages (DSLs) and Application Programming Interfaces (APIs) on a per-portal basis. The use of Web 3.0 standards enables data federation across multiple data sources, for instance using SPARQL’s *SERVICE* queries.

It is noteworthy that the road map engine described hosts comparatively little data, in contrast to other services, such as FIREHOSE (https://confluence.broadinstitute.org/display/GDAC/Home), which hosts versioned copies of the TCGA’s data. In contrast, the TCGA Roadmap described here seeks to provide a programmatic mechanism for computational resources to discover subsets of the TCGA’s data using standard languages, while leaving the hosting of the data itself to the TCGA. This decreases the cost of operation of the TCGA Roadmap, enabling increased sustainability of the effort.

The International Cancer Genome Consortium (ICGC) (Zhang *et al.*, 2011a) also makes the TCGA’s data available. The ICGC provides access to TCGA data in number of formats, such as FTP (ftp://data.dcc.icgc.org/), and through a variety of graphical query builder interfaces. Data may also be accessed through APIs such as SPARQL, Java, REST and SOAP, but doing so requires a user to maintain a local installation of BioMart (Zhang *et al.*, 2011b). The provided access modalities (FTP, graphical query builders or local installations) preclude the integration of ICGC data in bioinformatics tools delivered as web apps within the browser, which is a key use case of the TCGA Roadmap.

Memorial Sloan-Kettering Cancer Center’s cBio Cancer Genomics Portal (Cerami *et al.*, 2012), available at http://www.cbioportal.org/public-portal/, and its accompanying web API, provide another mirrored version of the data available in the TCGA. Their provision of a web API is a significant advancement in integration by allowing the use of query results in web-based bioinformatics tools. However, the use of a custom DSL for driving this API makes data federation between this and other sources cumbersome for software development by non-TCGA experts. As seen in the use cases, the TCGA Roadmap is explicitly tailored for use in federated queries.

### 4.7 Use cases

To illustrate possible applications of the TCGA Roadmap, we provide here a series of use cases demonstrating the unique capabilities of a standards-based approach. These use cases underscore the primary application of the TCGA Roadmap as a technical framework for the discovery of subsets of data files within the TCGA, and highlight the use of this framework in the creation of higher-level bioinformatics tooling.

**Federated queries**. A common task in any data-driven discipline is the integration (or federation) of data from multiple sources. In the case of the TCGA Roadmap, the SPARQL query language provides a natural mechanism for querying multiple databases simultaneously. For instance, using the *SERVICE* keyword in SPARQL 1.1, a user can query multiple SPARQL endpoints and integrate the results. An example query, at https://gist.github.com/4625146, demonstrates this by using the TCGA Roadmap to discover sample ID’s for the data files available, uses a second SPARQL endpoint to map sample ID’s to patient ID’s and finally uses a third endpoint to collect patient demographic information.

**Real-time dataset discovery**. We anticipate that much of the next generation of bioinformatics tools will be delivered in the same way as a significant portion of the current generation of consumer software: web apps in the browser. The delivery of bioinformatics tools as elements of such a computational ecosystem requires the ability to discover the particular subsets of public big data resources, such as the TCGA, as often the environment of this ecosystem (e.g. a web browser) may not have sufficient capacity to manage the multiple terabytes representing the entirety of the data available. As a particular analysis example, a reverse-phase protein analysis (RPPA) tool was developed (http://bit.ly/TCGA-RPPA), which enables real-time analysis of RPPA data from the TCGA in the Google Chrome web browser. This tool uses the road map presented here to sift through the data files available and select the URLs for those containing Level 3 RPPA results. These URLs are used to access the data files, which are then parsed within the browser and presented through a graphical user interface.

**DSL creation**. DSLs are specialized languages designed to make particular classes of problems more easily expressed. In the context of the TCGA Roadmap, a DSL eases the challenges associated with understanding the particular ontology used to model TCGA data files and crafting SPARQL queries to extract meaningful results from the Road map. Rather than beginning with a DSL for data exploration, as many data portals do, the TCGA Roadmap makes use of SPARQL to provide a foundation for the creation of diverse DSLs. To illustrate this point that a SPARQL endpoint is a more interoperable starting point to develop application-specific DSLs and practical APIs, a browser-based (JavaScript) DSL was created in the framework of a TCGA Toolbox for Google Chrome (http://bit.ly/TCGA-QL). Because the SPARQL endpoint remains exposed to HTTP calls, the enhancement of TCGA-QL for specific purposes or the development of an entirely different DSL on top of SPARQL is completely unhindered. In a nutshell, using SPARQL as an intermediate API to develop multiple DSLs maximizes the range of secondary uses of the same data. As shown in a video tutorial (http://youtu.be/_wCPZytFOk4), the development and use of a DSL to enable new functionalities in the operation of TCGA data is particularly convenient in Application ecosystems based of the web platform. As described in that screencast, TCGA-QL itself is worth a closer look as a DSL that enables and simplifies the migration of SPARQL’s query and data federation capabilities into the increasingly browser-based working environment of the bioinformatician.

## 5 CONCLUSIONS

In addition to its role as a cancer genomics research reference data resource, TCGA data resource is evolving toward becoming a de facto collection of reference patient–derived records for cancer, including patient demographics, outcomes and tissue images along with genomic and proteomic results. As a result, the necessity for programmatic and reproducible discovery and access of particular subsets of the data resource most relevant to a given study or patient has become urgent. This need for more effective programmatic interoperability, along with the increased delivery of novel analyses and computational tools as executable elements of a Web 3.0 ecosystem, provides motivation for our development of a RDF and SPARQL road map of the data files available in the TCGA. The resulting self-updating road map may be used by itself, in conjunction with other data sources, or embedded in a data analysis ecosystem. SPARQL was also found to offer a capable API for data analysis applications, overcoming the need for the download of the entire TCGA dataset via data portals. Furthermore, a TCGA DSL, TCGA-QL, was developed to illustrate the argument that SPARQL provides a more solid and interoperable foundation for DSL and API development. Finally, the lessons learned from the development of this RDF- and SPARQL-based road map were condensed into three specific recommendations for biomedical big data resources. If addressed, the recommended features would completely overcome the current obstacle of manually identifying and downloading individual files to analyze them, therefore adding TCGA to the web’s global data space.
